# Enhanced Heat Resistance of Acrylic Pressure-Sensitive Adhesive by Incorporating Silicone Blocks Using Silicone-Based Macro-Azo-Initiator

**DOI:** 10.3390/polym12102410

**Published:** 2020-10-19

**Authors:** Hee-Woong Park, Hyun-Su Seo, Kiok Kwon, Jung-Hyun Lee, Seunghan Shin

**Affiliations:** 1 Green Chemistry & Materials Group, Korea Institute of Industrial Technology (KITECH), Cheonan 31056, Korea; binufamily@kitech.re.kr (H.-W.P.); radiant@kitech.re.kr (H.-S.S.); kioks@kitech.re.kr (K.K.); 2 Department of Chemical and Biological Engineering, Korea University, Seoul 02841, Korea; leejhyyy@korea.ac.kr; 3 Department of Green Process and System Engineering, University of Science & Technology (UST), Daejeon 34113, Korea

**Keywords:** pressure-sensitive adhesive (PSA), silicone acrylic block copolymer, thermal stability, peel strength, loop tack

## Abstract

To improve the heat resistance of acrylic-based pressure-sensitive adhesive (PSA), silicone-block-containing acrylic PSAs (SPSAs) were synthesized using a polydimethylsiloxane (PDMS)-based macro-azo-initiator (MAI). To evaluate the heat resistance of the PSA films, the probe tack and 90° peel strength were measured at different temperatures. The acrylic PSA showed that its tack curves changed from balanced debonding at 25 °C to cohesive debonding at 50 °C and exhibited a sharp decrease. However, in the case of SPSA containing 20 wt% MAI (MAI20), the balanced debonding was maintained at 75 °C, and its tack value hardly changed with temperature. As the MAI content increased, the peel strength at 25 °C decreased due to the microphase separation between PDMS- and acryl-blocks in SPSA, but the shear adhesion failure temperature (SAFT) increased almost linearly from 41.3 to 122.8 °C. Unlike stainless steel substrate, SPSA showed improved peel strength on a polypropylene substrate due to its low surface energy caused by PDMS block. Owing to the addition of 20 wt% silicone-urethane dimethacrylate oligomer and 200 mJ/cm^2^ UV irradiation dose, MAI20 showed significantly increased 90° peel strength at 25 °C (548.3 vs. 322.4 gf/25 mm for pristine MAI20). Its heat resistance under shear stress assessed by shear adhesion failure test (SAFT) exhibited raising in failure temperature to 177.3 °C when compared to non-irradiated sample.

## 1. Introduction

Pressure-sensitive adhesive (PSA) is a semisolid material that adheres to various substrates at room temperature without additional chemical reactions and does not leave a residue after removal. Owing to these unique characteristics, it has been used in various industrial fields, such as packaging, labeling, medical, pharmaceutical, automotive, and electronics [1,2,3,4]. In general, PSA can be divided into three types: acrylic copolymers, polysiloxane, and rubber. Among these three types, acrylic PSA is the most widely used because it has advantages such as low cost, good resistance to light and oxygen, and optical clarity. However, acrylic PSA also has disadvantages, such as low adhesion to low surface-energy substrates and low heat resistance. In particular, the low heat resistance of acrylic PSA is an obstacle to expanding its application [4,5].

In recent years, there has been a growing demand for PSA with improved thermal stability for special applications, such as thermal conductive tapes in electronics and automotives [6]. As a heat-resistant PSA, silicone-type PSA is commonly used because silicone-type PSA has low surface tension, high chemical/weather resistance, and high temperature resistance (−60 °C to 250 °C) owing to the highly flexible Si-O-Si bond. However, it has lower adhesion properties than acrylic PSA in most cases and is expensive [7].

In addition to silicone-type PSA, various methods for improving the thermal stability of acrylic PSA have been proposed. The first method to enhance the thermal stability of acrylic PSA is to improve the cohesion force through molecular design. A typical example is to suppress the mobility of acrylic polymer chains by a crosslinking reaction [8,9,10,11]. Chen et al. designed a triazine heterocyclic monomer and copolymerized it with an acrylic monomer to prepare a crosslinked PSA tape to improve its heat resistance [9]. Pang et al. dispersed reactive nanosilica to acrylic PSA and increased thermal stability by crosslinking through UV irradiation [10]. Joo et al. prepared a semi-interpenetrating polymer network (semi-IPN) structure in PSA tape using trifunctional acrylic monomers of different lengths, followed by UV irradiation. They reported greatly improved heat resistance [11]. However, the crosslinking method also reduces adhesion properties, such as peel strength and tack, by suppressing the mobility of the acrylic chain.

Another method is to incorporate siloxane units into the acrylic PSA. However, due to poor compatibility between silicone and organic chains, simple blending induces macroscopic phase separation that results in property changes with time. Accordingly, it is difficult to obtain good adhesion characteristics with this method. To overcome this problem, a number of methods have been developed: a silicone adhesive is blended with an aqueous acrylic adhesive using an emulsifier [12], a reactive silicone resin-type seed is used for emulsion polymerization with an acrylic monomer [13], or a prepolymer, which is prepared with functionalized polydimethylsiloxane (PDMS), is introduced into the acrylic chain via copolymerization [14]. In this regard, a PDMS-based macro-azo-initiator (MAI) was developed to easily introduce a silicone unit into a polymer chain [15,16]. The PDMS-containing block copolymer changes the surface and bulk properties due to the unique characteristics of PDMS. This approach has been used in combination with various polymers, such as polystyrene [17,18], polyamide [19], and poly(methyl methacrylate) [20,21]. However, to the best of our knowledge, there are very few such studies related to PSA.

In this work, we synthesized silicone block-containing acrylic PSAs (SPSAs) using MAI to improve the thermal stability of acrylic PSA. The average molecular weight and basic properties of SPSAs prepared with different MAI contents were measured. To evaluate the heat resistance of the SPSA films, their probe tack and 90° peel strength were assessed with increasing temperature, and their shear adhesion failure temperature was also measured. The 180° peel strength of SPSA was measured on stainless steel (SUS) and polypropylene (PP) substrates to evaluate the effect of silicone block on the peel strength. To improve the adhesion properties of SPSA, silicone-urethane dimethacrylate (SiUDMA) oligomer was added to enhance the compatibility, and then UV curing was performed. The adhesion properties and thermal stability of SiUDMA-modified SPSA were evaluated according to the two above-mentioned factors.

## 2. Experimental

### 2.1. Materials

**Figure 1:**
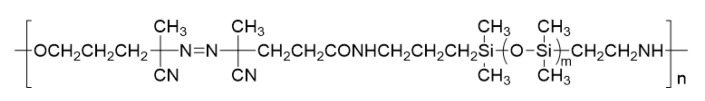
Chemical structure of macro-azo-initiator (MAI) (VPS-1001).

PDMS-based macro-azo-initiator (MAI, VPS-1001) was purchased from Wako Pure Chemical Industries, Ltd. (Osaka, Japan), and its structure is given in Figure 1. Its average molecular weight is 82,300, the molecular weight of the PDMS unit is approximately 10,000 and the content of azo groups is approximately 0.1 mmol/g. Azobisisobutyronitrile (AIBN, 98%) was purchased from Jusei Chemical Co., Ltd. (Tokyo, Japan). Carbinol-terminated PDMS (KF6000, Mw = 935, hydroxyl value = 120 mg KOH/g) was purchased from Shinetsu Co., Ltd. (Tokyo, Japan). Dibutyltin dilaurate (DBTDL, 95%), isophorone diisocyanate (IPDI, 98%), 2-ethylhexyl acrylate (2-EHA, 98%), acrylic acid (AA, 99%), isobornyl acrylate (IBA, >85%), 2-hydroxyethyl methacrylate (HEMA, 99%), and ethyl acetate (EA, 99.8%) were purchased from Aldrich (MO, USA). Irgacure 907 (2-Methyl-4-methylthio-2-morpholinopropiophenone, 98%) was supplied by BASF Co., Ltd (Ludwigshafen, Germany). and used as a photoinitiator (Germany). All chemicals were used without further purification.

### 2.2. Synthesis of Silicone Block-Containing Acrylic PSA (SPSA)

**Scheme 1:**
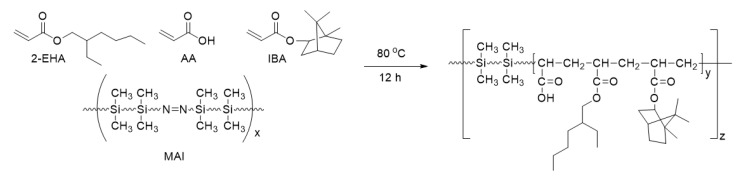
Synthesis of silicone block-containing acrylic pressure-sensitive adhesive (PSA) (SPSA).

SPSA was synthesized by solution polymerization using MAI (Scheme 1). The weight ratio of acrylic monomer was fixed to 2-EHA:AA:IBA = 75:5:20, and the composition was changed according to the amount of initiator (see Table 1). Control PSA was synthesized using AIBN, a general thermal initiator. Acrylic monomers (2-EHA, AA, IBA) were dissolved in EA to an overall concentration of 40 wt% and added to a 2-neck round bottom flask equipped with a reflux condenser. Then, the temperature was raised to 80 °C, and the mixture was stirred for 5 min under N_2_ atmosphere. The initiator was diluted in solvent (40% concentration) and added dropwise. After 12 h, to complete the reaction, the mixture was cooled to room temperature, and solvent was added to adjust the solid content to 40%. In Table 1, sample code means SPSA synthesized using different amount of MAI (e.g., MAI5 means SPSA synthesized with 5 wt% of MAI).

### 2.3. Synthesis of Silicone Urethane Dimethacrylate (SiUDMA)

**Figure 2:**
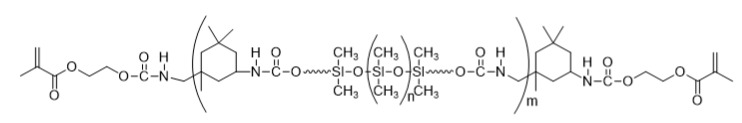
Chemical structure of silicone urethane dimethacrylate (SiUDMA).

SiUDMA was synthesized based on our previous work [22], and its chemical structure is given in Figure 2. IPDI (17.83 g, 80 mmol) and DBTDL (0.07 g, 0.1 wt% of total mass) were added to a 250-mL 2-neck round bottom flask and stirred for 15 min at 50 °C under N_2_ atmosphere. Reactive PDMS, KF6000 (50 g, 53 mmol) was slowly added dropwise over 20 min, followed by stirring for 40 min. To introduce UV cross-linkable functionality, HEMA (3.48 g, 27 mmol) was added dropwise over 20 min, stirred for 40 min, and then cooled to room temperature to complete the reaction. The number- and weight-average molecular weights of SiUMDA were 4800 and 7500, respectively. The solution viscosity was 3199 ± 212 mPas (50 wt% in EA).

### 2.4. Preparation of PSA Tape Samples

A photoinitiator (Irgacure 907) was added in 0.1 part to solutions of PSA. To prepare PSA films, solutions of the control PSA and SPSA were spread onto corona-treated polyethylene terephthalate (PET) films using a Baker applicator, and the films were placed in a convection oven at 80 °C for 30 min to remove the residual solvents. After drying, the film thickness measured with a vernier caliper was approximately 50 μm.

To modify the adhesion properties of SPSA, UV irradiation was conducted with SiUDMA oligomer. When SiUDMA was added at 10, 20, 30, 40 wt% relative to solid content of MAI20, the highest 180° peel strength was obtained at 20 wt% (see Figure S1 in Supplementary Materials). Accordingly, SiUDMA content was fixed at 20 wt% and UV irradiation (200 and 1000 mJ/cm^2^) was applied to induce a crosslinking reaction after drying. The compositions of SiUDMA-modified SPSA samples are presented in Table 2.

### 2.5. Measurements

The number- and molecular-average molecular weights (*M_n_* and *M_w_*) and polydispersity index (PDI) for each of PSA and SiUDMA were measured using gel permeation chromatography (GPC, PL-GPC 220, Agilent Technologies, Santa Clara, CA, USA). The samples were dissolved in a tetrahydrofuran solution at a concentration of 5 wt%, and the operation temperature was 30 °C.

Fourier transform infrared (FTIR) spectra of PSAs were recorded using a NICOLET 6700 (Thermo Fisher Scientific, Waltham, MA, USA). PSA solution was coated on a PET film and then dried at 80 °C for 1 h to form a 50-μm film. Attenuated total reflection (ATR) mode was used with a diamond prism and a 45° incident angle. The measurement range was 650–4000 cm^−1^, and the resolution was 4 cm^−1^.

Contact angle measurement was performed using a contact angle goniometer (Phoenix 300, Surface & Electro-Optics, Suwon, Korea). Distilled water and diiodomethane were dropped on the PSA film, and the contact angle was recorded after 5 s. The process was repeated three times at 23 ± 2 °C. Based on the measured contact angle, the surface energy of PSAs was calculated by the Owens–Wendt method [23].

The solution viscosities of the PSAs were measured using a Brookfield DV-II (Middleboro, MA, USA) at 25 °C. The solid contents of all samples were fixed at 40 wt%. For temperature stabilization, the samples were placed in a viscometer for 20 min. Then, the viscosities of the samples were measured by selecting the suitable rpm and spindle.

The storage modulus (G’) and loss tangent of PSAs were measured using a rheometer (MCR 102, Anton Paar, Graz, Austria). The samples were mounted on an 8-mm-diameter round plate, and the gap between the plates was 0.5 mm. The plates were twisted under conditions of 1% strain and 1-Hz frequency. The temperature range was from −40 to 80 °C with a 10 °C/min heating rate.

The gel fraction of each PSA was obtained by an extraction method using metallic paper (20 mesh). The dried PSA sample (1 g) was wrapped with metallic paper and then put in a 40 °C EA solution and stirred for 24 h. The remaining solid in the metallic paper was filtered and dried in an 80 °C vacuum oven until a constant weight was obtained. The gel fraction was calculated by comparing the extracted sample weight to the initial sample weight.

The haze of each PSA film was measured using the HZ-V3 haze meter (Suga, Japan). After the baseline was measured using an uncoated PET film, the hazes of the PSA films were measured.

The probe tacks, loop tacks, 90° peel and 180° peel strengths of PSA films were measured using a SurTA system (ChemiLab, Suwon, Korea). For probe tack measurement, 60-μm-thick PSA coating a SUS (type 304) plate was mounted on the bottom grip of the instrument. The temperature of the plate was set at 25, 50, 75, and 100 °C. The probe diameter was 1/8 inch. The debonding speed was 0.5 mm/s, and the maximum debonding load value was recorded.

For 90° peel strength measurement, PSA films were prepared by cutting into a size of 25 mm × 60 mm. The samples were attached to the SUS, rolled twice using a 2-kg rubber roller, and left for 20 min. After that, the sample was mounted on the instrument, and the temperature was set at 25, 50, 75, and 100 °C. The peeling speed was 300 mm/min.

The loop tack of the PSA film was evaluated by the ASTM D6195 method. The dried PSA film was prepared in a size of 25 mm × 100 mm. The sample was inserted into the upper grip in a loop shape to expose the adhesive side, and the substrates were inserted into the bottom grip. SUS and PP were used as substrates, the surface energies of which were 46.1 and 32.5 mN/m, respectively. When the contact area between the film and the substrate reached 25 mm × 25 mm, the looped PSA film was raised at a speed of 0.5 mm/s, and the maximum force was recorded.

To measure the 180° peel strength, a PSA film was prepared with a size of 25 mm × 60 mm, attached to the substrates and rolled twice with a 2-kg rubber roller. The peeling speed was 300 mm/min.

The shear adhesion failure test (SAFT) was performed to evaluate the heat resistance and shear strength of the PSA films. Each specimen (25 mm × 25 mm) was attached to the SUS and left at 25 °C for 24 h. A load was applied using a 1-kg weight, and each sample was placed in an oven and heated to 25–200 °C at a rate of 0.5 °C/min.

## 3. Results and Discussion

### 3.1. Synthesis and Characterization of SPSAs

The SPSAs were synthesized using MAI, and their basic properties are displayed in Table 3. As the MAI content increased, the molecular weight of SPSA decreased, and its PDI value increased. This is well understood by considering that the molecular weight of acrylic PSA decreases and its PDI value increases with the concentration of azo-initiator [1,20,24]. In particular, MAI30 showed a rapid increase in solution viscosity during polymerization. This is due to the gelation caused by the excessive reaction heat with high MAI concentration. The gel fraction of MAI30 was calculated to be 15.84%. As a result of gel formation, the SPSA becomes non-homogenous and its molecular mass is most likely determined only for the smaller molecules of polymer dissolved in solvent, not for the polymer in gel-like state dispersed in solvent. Meanwhile, MAI5 showed a higher molecular weight and viscosity than control PSA, although two samples have the same concentration of azo group. This is because MAI is a macro-azo initiator whose average molecular weight is 82,300 g/mol, which is much larger than that of AIBN.

**Figure 3:**
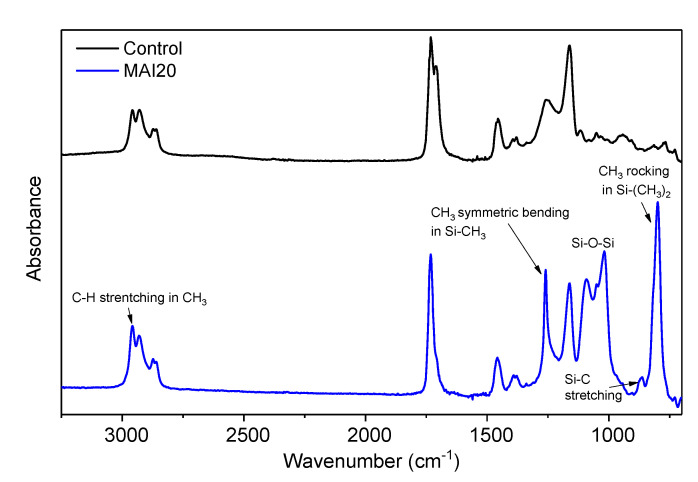
FTIR spectra of Control and MAI20 PSAs.

Figure 3 shows the FTIR spectra of SPSA and control PSA. The reaction proceeded well because the C=C stretching peak at 1640 cm^−1^ was not observed. Aside from control PSA, PDMS-related peaks were observed in SPSA: CH_3_ stretching at 2960 cm^−1^, CH_3_ deformation at 1260 cm^−1^, Si–O–Si stretching, and deformation at 1100 and 1016 cm^−1^, Si–C stretching at 860 cm^−1^, and CH_3_ rocking in Si–(CH_3_)_2_ at 800 cm^−1^.

### 3.2. Adhesion Property and Thermal Stability of SPSA

**Figure 4:**
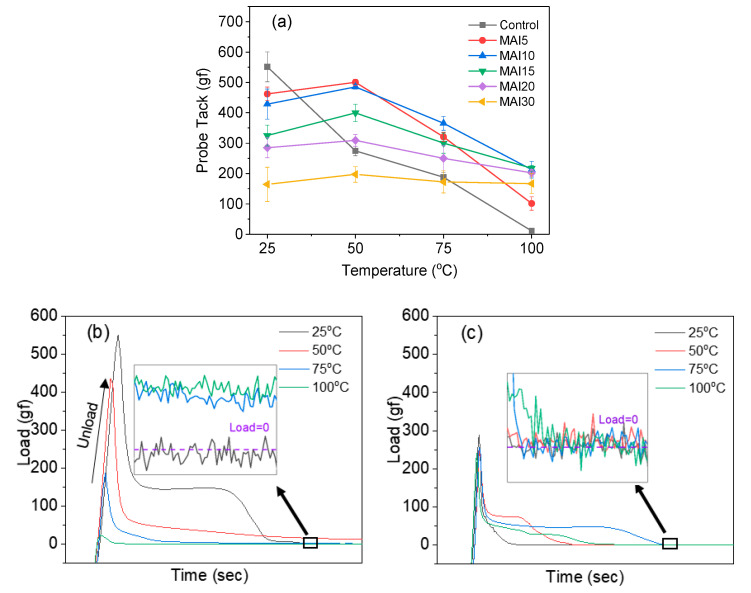
Probe tack of SPSAs as a function of temperature (**a**), probe tack curves of control (**b**), and MAI20 (**c**).

Figure 4a shows the probe tack of various SPSAs as a function of temperature. The probe tack of control PSA decreased rapidly with temperature. However, the probe tack of SPSAs decreased less steeply with temperature compared to control PSA. Moreover, they changed much less with temperature if the MAI content was more than 20 wt% (MAI20 and MAI30).

Figure 4b,c shows the unloading behavior of the probe tack of the control and MAI20 PSAs with different temperatures. The control PSA showed a plateau after debonding at 25 °C, the load rapidly decreased to 0, and no residue was found on the probe. However, as the temperature increased to 50 °C, liquid-like behavior began to appear, and high elongation without a plateau was observed. The residue was left on the probe. According to Gdalin et al., the types of tack curves are largely divided into three categories, such as balanced, cohesive, and solid-like debonding, depending on the balance between cohesive force and free volume of PSA [25].

The behavior of control PSA at 25 °C is “balanced debonding,” which occurs when cohesion force and free volume are balanced. The behavior above 50 °C is expressed as “cohesive debonding,” which occurs in a liquid-like adhesive with low cohesive force. This means that the cohesive force of the control PSA decreases sharply but the free volume increases with temperature. In addition, at 50 °C or above, the load does not become zero, and elongation continues. This means that, although the cohesive force of PSA decreases, its adhesion strength increases due to the improved surface wetting resulting from the viscosity lowered by temperature (see the inset of Figure 4b). Alternately, MAI20 showed similar probe tack curves at 50 °C or above, which was different from the control PSA. As the temperature increased, the maximum force value decreased somewhat, but there still remained a plateau, and the final load became zero (see the inset of Figure 4c). This means that MAI20 belongs to optimized adhesion (balanced debonding) and has superior heat resistance to control PSA.

**Figure 5:**
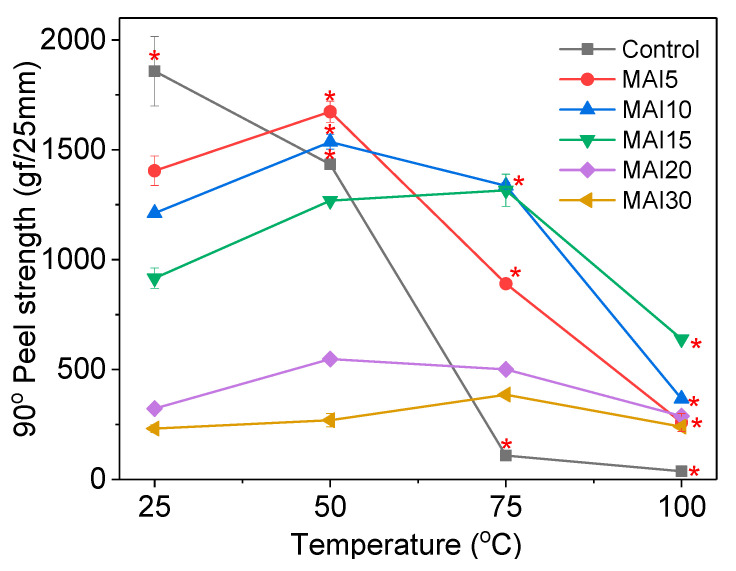
90° peel strength of various SPSAs as a function of temperature. The red asterisk denotes cohesive failure.

**Figure 6:**
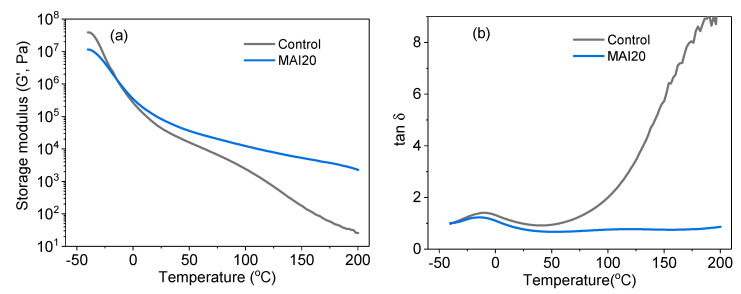
Storage modulus (G’) (**a**) and tan δ (**b**) of control PSA and SPSA (MAI20).

Figure 5 shows the 90° peel strength of SPSAs as a function of temperature. The trend was similar to that of the probe tack. In the MAI20 and MAI30 samples, cohesive failure did not appear until 100 °C, and their peel strength did not change significantly with temperature, although their initial values were relatively small. To explain this, the viscoelastic properties of the control PSA and MAI20 were examined and are displayed in Figure 6.

Figure 6 shows that the storage modulus (G’) of control PSA decreased sharply with temperature, and the slope changed and became almost constant at approximately 100 °C, indicating that melting occurred. Alternately, MAI20 showed relatively lower storage modulus variation with temperature compared to control PSA (25 °C/100 °C = 86,841/12,280 vs. 25 °C/100 °C = 45,992/2397). This helps to understand the relatively small changes in the probe tack and 90° peel strength of MAI20 with temperature. In the case of tan δ (see Figure 6b), the glass transition temperature (*T_g_*) of MAI20 was lower than that of control PSA (−13.86 °C vs. −9.86 °C), which was due to the contribution of higher flexibility of PDMS to the MAI20 flexibility on molecular level.

**Figure 7:**
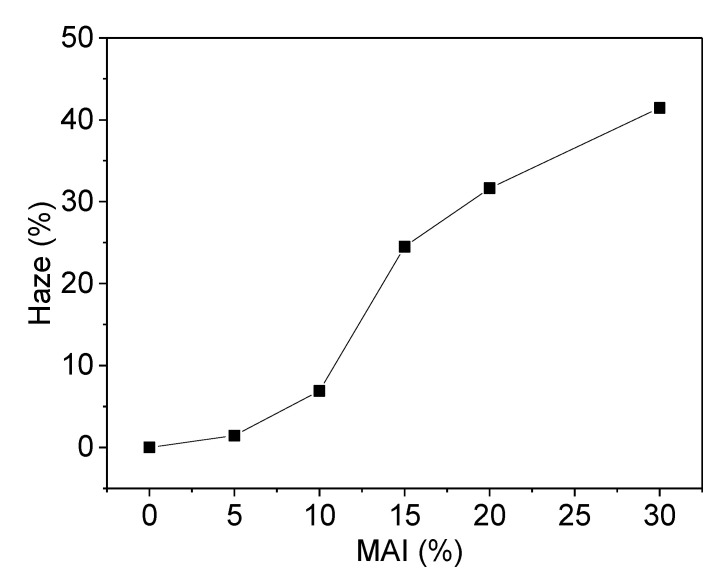
Haze of SPSA films with MAI content.

In the case of 90° peel strength at 25 °C, they decreased significantly with MAI content (see Figure 5). This is due to the microphase separation between the PDMS and acrylic blocks in the SPSA polymer chains. This microphase separation can be estimated by measuring the haze of SPSAs, as shown in Figure 7. The haze of SPSAs increased steeply according to the MAI content, which means that microphase separation was induced by MAI. The effect of phase separation on the adhesion properties of PSA was well studied by Kim et al. [26,27,28]. They found that the tack, peel strength, and shear strength of adhesives decrease sharply by phase separation. Therefore, the compatibility of SPSAs should be improved to overcome this problem, which will be discussed in Section 3.4.

**Figure 8:**
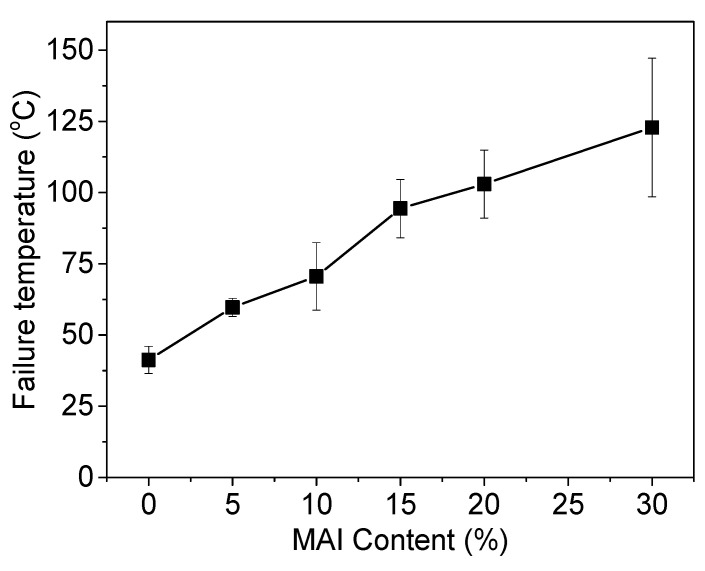
Shear adhesion failure test (SAFT) results on SPSA influenced by MAI content.

A shear adhesion failure test (SAFT) was also performed to evaluate the shear strength and heat resistance characteristics of the SPSA (Figure 8). The failure temperature increased linearly according to the PDMS content in the adhesive. Although the molecular weight of SPSA decreased slightly with MAI content, the improvement of heat resistance by PDMS was prevalent.

### 3.3. Adhesion Property of SPSA on SUS and PP

**Figure 9:**
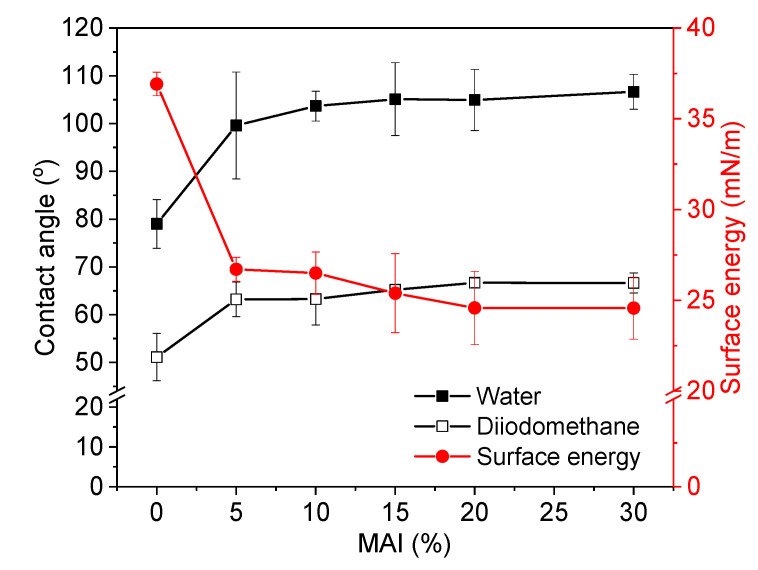
Water and diiodomethane contact angle and surface energy of SPSAs as a function of MAI content.

Figure 9 shows the water and diiodomethane contact angles and surface energies of SPSAs synthesized with different amounts of MAI. SPSAs showed a high contact angle and low surface energy compared with control PSA. However, the changes in contact angle and surface energy with increasing MAI content were not so large. This means that most of the surface of the SPSA film is saturated with the PDMS chain even with a small amount of MAI. This result can be understood by referencing the work of Inoue et al. [29]. They reported that the water contact angle of a poly(dimethylsiloxane-b-methyl methacrylate) block copolymer was higher than 100° even with 1–2% PDMS content. They found that, if the molecular weight of the PDMS block is over 2000, the block copolymer has a PDMS-rich surface even at a small amount of PDMS. The PDMS chain of MAI is long enough (*M_n_* = approximately 10,000) for this phenomenon to occur.

**Figure 10:**
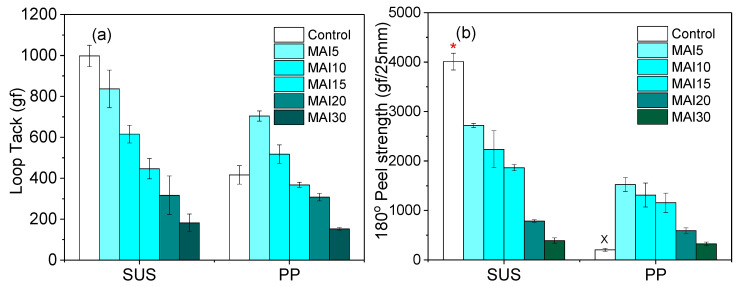
Loop tack (**a**) and 180° peel strength (**b**) of SPSA on stainless steel (SUS) and polypropylene (PP) substrates. The red asterisk denotes cohesive failure, and x denotes stick-slip behavior.

Owing to the low surface energy, SPSA showed improved adhesion properties on the low surface energy substrate. Figure 10 shows the loop tack and 180° peel strength of SPSAs measured on SUS and PP. Compared with control PSA, the SPSA series showed remarkably higher peel strength on PP.

The control PSA showed cohesive failure on SUS, whereas it showed stick-slip behavior on PP. Stick-slip is known to occur if the strength of the adhesive bond (*σ_a_*) is similar to the cohesive strength of the PSA (*σ_c_*) [30]. As a result that the surface energy of control PSA is higher than that of PP (36.9 vs. 32.5 mN/m), *σ_a_* of control PSA on PP will decrease and become close to *σ_c_*. Meanwhile, SPSA showed that the tack and peel strength decreased with MAI content. This is due to the microphase separation between the two incompatible blocks (PDMS and acrylic blocks) in MAI, as explained previously.

### 3.4. Adhesion Property Modification of SPSA by SiUDMA

As displayed in Figure 5, MAI20 and MAI30 had overly low 90° peel strength at 25 °C with maintaining this level over the entire temperature range. To improve the adhesion properties of these SPSAs, SiUDMA oligomer was added, and UV irradiation was conducted. SiUDMA was added to MAI20, which showed high thermal stability, and the detailed properties of SiUMDA-modified MAI20 are given in Table 4. By the addition of SiUDMA, the solution viscosity (40 wt% solid in EA, 25 °C) of MAI20 was reduced from 58,300 mPas to 25,500 mPas by the low viscosity of SiUDMA, and the film haze also decreased from 34.65% to 18.34% due to partial suppression of phase separation [22].

**Figure 11:**
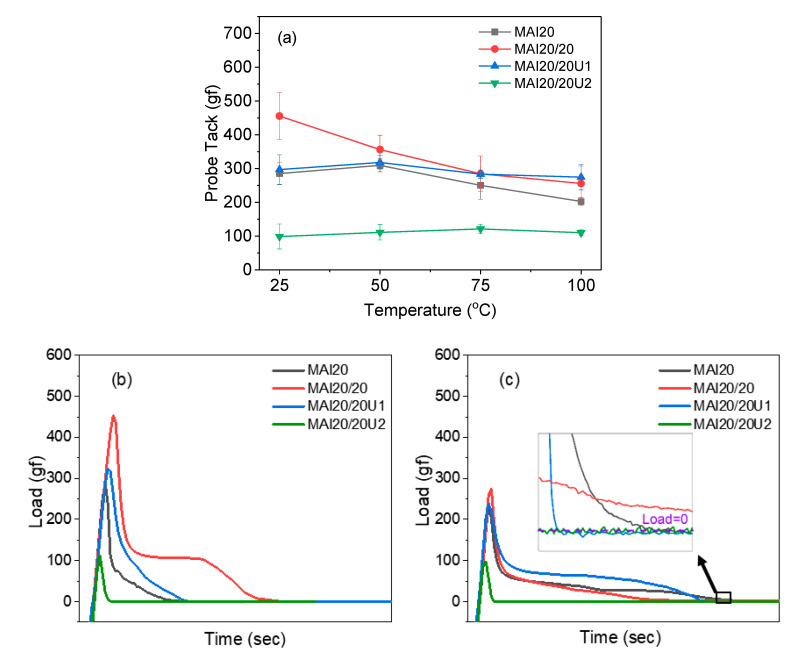
Probe tack of SiUDMA-modified MAI20 as a function of temperature (**a**), probe tack curves at 25 °C (**b**), and 100 °C (**c**).

Figure 11 shows the unloading behavior of the tack of SiUDMA-modified MAI20. In the case of MAI20/20, the tack increased significantly, and a plateau appeared after the maximum peak (Figure 11a,b). SiUDMA induced balanced debonding by reduced cohesive force and increasing the adhesive property. UV irradiation decreased the tack and plateau regions by crosslinking. As the temperature increased to 100 °C (Figure 11c), high elongation was observed in MAI20/20, and tack did not immediately drop to zero, which means that MAI20/20 behaved like a liquid-like adhesive, as explained in Section 3.2. The UV crosslinked samples exhibited balanced or solid-like debonding tack behavior at 100 °C depending on the UV dose exemplified for the two dose values.

**Figure 12:**
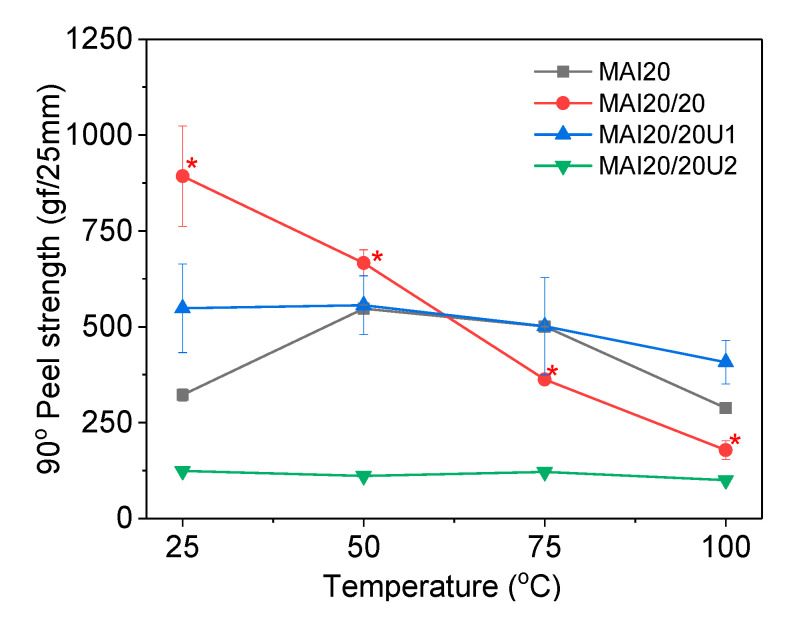
90° peel strength of the SiUDMA-modified MAI20 series as a function of temperature. The red asterisk denotes cohesive failure.

Figure 12 shows the 90° peel strength of the SiUDMA-modified MAI20 series. MAI20/20 showed improved peel strength compared to MAI20 (892.9 vs. 322.4 gf/25 mm), but the failure mode changed from adhesive to cohesive and the peel strength steeply decreased with temperature. By the 200-mJ/cm^2^ UV dose (MAI20/20U1), the peel strength at 25 °C decreased but was still higher than that of MAI20 (548.3 vs. 322.4 gf/25 mm). MAI20/20U1 showed thermally stable peel strength until 100 °C. However, a UV dose of 1000 mJ/cm^2^ reduced the peel strength too much but improved the thermal stability.

**Figure 13:**
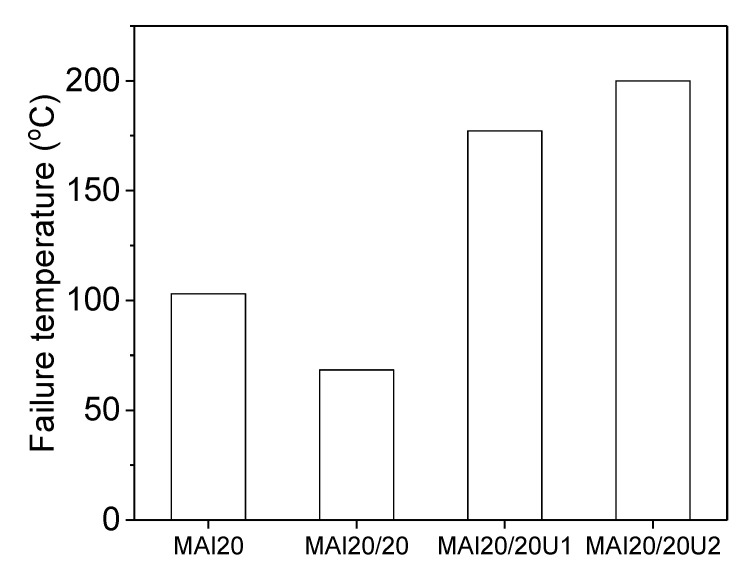
SAFT result on the SiUDMA-modified MAI20 series.

The SAFT of the SiUDMA-modified MAI20 series (Figure 13) showed that the failure temperature of MAI20/20 decreased because of the plasticizing effect due to the low molecular weight of SiUDMA. However, the UV-irradiated sample significantly improved the failure temperature via the formation of a semi-IPN structure. As displayed in Table 4, depending on the UV dose, the gel fraction was increased from 0% to 28.43% (200 mJ/cm^2^) and 88.21% (1000 mJ/cm^2^). This indicates that a crosslinking reaction occurred in this system.

## 4. Conclusions

Silicone block-containing acrylic PSAs (SPSAs) were synthesized using a PDMS-based macro-azo-initiator (MAI) to improve the adhesion properties of acrylic PSA at an elevated temperature, and the following conclusions were obtained.

SPSAs can be easily synthesized by incorporating PDMS blocks into the acrylic PSA using MAI. SPSA shows a higher viscosity than PSA synthesized using AIBN containing the same concentration of azo group. As the content of MAI increases, the molecular weight of SPSAs decreases, and the molecular weight distribution becomes broader.

Due to the PDMS blocks, SPSAs exhibit relatively small changes in tack and peel strength with temperature compared to acrylic PSA because the storage modulus of SPSA gradually decreases with temperature. Furthermore, SPSA with a high MAI content maintains balanced debonding even at a relatively high temperature.

The surface of SPSA is almost saturated with PDMS chains even at a small amount of MAI because of the large size of PDMS in MAI. Accordingly, the surface energy of SPSA changes slightly with MAI content. Owing to its low surface energy, SPSA shows improved adhesion properties on PP, a low-surface-energy substrate.

However, SPSAs have lower tack and peel strength than acrylic PSA at 25 °C due to the microphase separation between PDMS and acrylic blocks. The microphase separation in SPSA is leveled by the addition of SiUDMA oligomer, and 90° peel strength at 25 °C increases with the failure mode change from adhesive to cohesive. However, the tack and peel strength of SPSA decrease rapidly with temperature due to the plasticizing effect of SiUDMA. Through the formation of the semi-IPN structure by an appropriate UV irradiation dose, it is possible to minimize tack and peel strength reduction and improve thermal stability.

## Figures and Tables

**Table 1:** Monomer composition of SPSA.

Sample Code	2-EHA		AA		IBA		Initiator	
	g	mmol	g	mmol	g	mmol	g	Azo Group (mmol)
Control *	75.00	398.85	5.00	68.70	20	81.62	0.084	0.50
MAI5	71.25	378.91	4.75	65.26	19	77.53	5.00	0.50
MAI10	67.50	358.96	4.50	61.83	18	73.45	10.00	1.00
MAI15	63.75	341.02	4.25	58.39	17	69.37	15.00	1.50
MAI20	60.00	319.08	4.00	54.95	16	35.29	20.00	2.00
MAI30	52.50	279.19	3.50	48.08	14	57.13	30.00	3.00

* AIBN was used instead of MAI.

**Table 2:** Formulation of UV-cured SPSA composition.

Sample Code	MAI20 * (g)	SiUDMA (g)	EA (g)	UV Dose (mJ/cm^2^)	
MAI20/20	10 (4/6)	1.0	1.5	-	-
MAI20/20U1	10 (4/6)	1.0	1.5	200	-
MAI20/20U2	10 (4/6)	1.0	1.5	-	1000

* The solid content of MAI20 is 40 wt% (EA is 60 wt%).

**Table 3:** Basic information on synthesized SPSAs.

	*M_n_*/*M_w_*/PDI	Solution Viscosity (40%, mPas)	Gel Fraction (%)
Control	127,000/301,000/2.37	1601	0.00
MAI5	164,900/414,000/2.51	69,600	0.00
MAI10	151,000/409,000/2.71	61,300	0.00
MAI15	94,300/373,000/3.95	58,800	0.07
MAI20	83,000/337,000/4.06	58,310	0.53
MAI30	49,900/248,400/4.97	721,000	15.84

**Table 4:** Detailed information on SiUDMA-modified MAI20.

Sample	SiUDMA (wt%)	Solution Viscosity (40 wt%, 25 °C, mPas)	Gel Fraction (%)	Film Haze (%)	UV (mJ/cm^2^)	
MAI20	0	58,300	0.53	34.65	-	-
MAI20/20	20	25,500	0.12	18.34	-	-
MAI20/20U1	20	-	28.43	16.14	200	-
MAI20/20U2		-	88.21	15.12	-	1000

## Supplementary Materials

The following are available online at https://www.mdpi.com/2073-4360/12/10/2410/s1, Figure S1. 180° peel strength of SiUDMA modified MAI20 PSAs on SUS and PP substrates. The red asterisk denotes cohesive failure.
