# Supplementary material for: Enhanced Heat Resistance of Acrylic Pressure-Sensitive Adhesive by Incorporating Silicone Blocks Using Silicone-Based Macro-Azo-Initiator
Source: Polymers (Basel). 2020 Oct 19;12(10):2410. doi: 10.3390/polym12102410 (PMC7650628; doi:10.3390/polym12102410)
Supplement: Supplementary file 1 [file polymers-12-02410-s001.zip › polymers-961066-supplementary.docx]

Supplementary Information

Enhanced heat resistance of acrylic pressure-sensitive adhesive by incorporating silicone blocks using silicone-based macro-azo-initiator

Hee-Woong Park, Hyun-Su Seo, Kiok Kwon, Jung-Hyun Lee and Seunghan Shin*


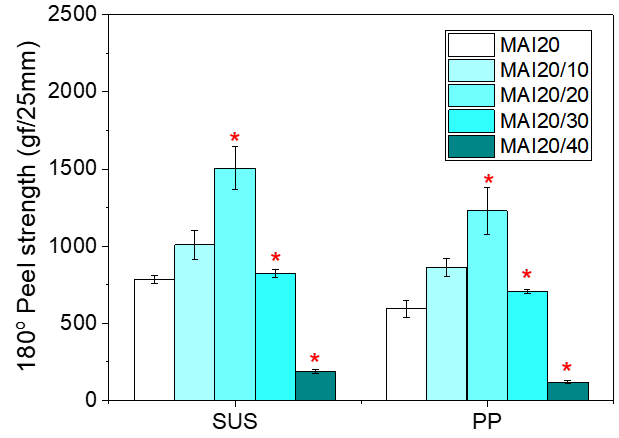


Figure S1. 180° peel strength of SiUDMA modified MAI20 PSAs on SUS and PP substrates. The red asterisk denotes cohesive failure.
